# Association of autonomic regulation with pain and disability in patients with chronic neck pain: a systematic review

**DOI:** 10.1590/1806-9282.20240597

**Published:** 2024-12-20

**Authors:** Al Afshan, Iram Iram, Tarushi Tanwar, Sana Rehman, Lubna Zahid, Zubia Veqar

**Affiliations:** 1 Jamia Millia Islamia, Centre for Physiotherapy and Rehabilitation Sciences – New Delhi, India.

## BACKGROUND

Globally, the prevalence rate of chronic neck pain (CNP) is 37%, with a higher occurrence observed among females of all age groups, higher-income countries, and sedentary office-based workers, compared to males of all age groups, lower-income countries, and physically active populations, respectively^1^. It is ranked fourth in terms of years lived with a disability and impaired quality of life^2^. It is a multifactorial condition with a sparsely known etiology and pathophysiology. Multiple population-based studies have identified psychosocial and psychophysiological factors as predictors of CNP^3,4^, indicating their significance in the development and maintenance of chronic pain conditions^5^. Among psychophysiological factors, autonomic nervous system (ANS) dysregulation is recognized as an important phenomenon in the development and maintenance of chronic musculoskeletal pain^5^. Therefore, assessment and knowledge of autonomic modulation are important when dealing with CNP.

Recent research has shown that CNP is linked to altered basal autonomic modulation along with altered autonomic reactivity to physical and mental stressors^6,7^. Furthermore, this altered regulation can cause a decrease in blood flow to the muscles and reduce oxygenation levels, potentially affecting physical functioning, contributing to increased disability levels, and hindering participation in physical activities. Thus, it exacerbates pain, deteriorates both physical and mental health, and impairs the overall quality of life^8^. However, conflicting findings have also been reported, with no discernible difference in basal autonomic regulation and reactivity to laboratory stressors^5,9,10^. Therefore, it is imperative to assess ANS dysregulation in patients with CNP and ascertain its association with pain intensity and the perceived level of disability. Consequently, this systematic review was conducted to investigate the existing literature on the association between ANS regulation and pain and disability in patients with CNP.

## METHODS

### Search strategy and information sources

The systematic review was comprehensively registered in PROSPERO in December 2023 (CRD42023490989). Preferred Reporting Items for Systematic Reviews and Meta-analyses (PRISMA) guidelines for reporting systematic reviews were stringently pursued^11^. For this purpose, an extensive search of five electronic databases, namely Medline, Web of Science, Scopus, ScienceDirect, and CENTRAL, and a reference list of the selected articles were performed. No restrictions on the start date were applied in the literature search. Only the full-text English reports of observational studies completed before November 2023 were included.

### Study selection

The following inclusion criteria were defined: (1) observational studies, (2) male and female ≥18 years, (3) CNP ≥3 months and assessing ANS regulation, and (4) pain and disability as outcomes.

Other types of studies, such as experimental design, reviews, pilot studies, and letters to the editor, that did not meet the inclusion criteria were excluded.

The articles retrieved by the search were imported into Zotero software for identification and duplicate exclusion. Subsequently, all the studies were individually screened for eligibility by two authors (AA and II), and any potential discrepancies were settled by the consensus of all authors.

### Data extraction

The data collected included methodological characteristics (study design, pain chronicity, sample characteristics, and related outcome variables), and the main findings are described in Table 1.

**Table 1:** Methodological characteristics and main findings of included studies.

Author Country	Duration (m)	Sample characteristics			ANS assessment methods	Outcomes		Main findings
		n (Female/Male), age (years)				p	D	
		CNP (f/m) Age; m (SD)		CON (f/m) Age; m (SD)				
Hallman et al.^6^ Sweden	≥ 6 m	n=45 (41/ 4), 20–50			HRV linear HR and BP, MBF	Borg CR10	NDI	Pain intensity showed a negative relation to SDNN (r=-0.435) and a positive relationship to resting BP (systolic: r=0.476) during rest. Disability correlated negatively with resting LFnorm HRV. Higher disability related to increased change in LFnorm reactivity to HGT.
		23 CNP (21/2) 40.5 (7.1)		22 CON (19/2) 40.8 (7)				
Kang et al.^17^ Taiwan	≥ 6 m	n=121 (91/30), 41.2 (31.9)				VAS	NDI	SDNN (r=-0.410), RMSSD (r=-0.322), and pNN50 (r=-0.343) showed a significant negative correlation with NDI scores (p<0.001, for all). Decreased LF (r=-0.230, p=0.011) and HF (r=-0.211, p=0.02) were also significantly associated with higher NDI scores.
		35 (29/6) Group 1 27.3 (9.1)	62 (62/0) Group 2 49.1 (9.3)	24 (0/24) Group 3 41.1 (13.6)				
Zaproudina et al.^16^ Finland	≥ 3 m	n=71 (42/29)				VAS	NDI	Pain intensity is significantly correlated with skin surface temperature in CNP patients with unilateral symptoms (ꞵ =0.441, p=0.024). No significant association between NDI with skin surface temperature.
		60 CNP (36/24) 30–49; 40.7 (5.9)		11 CON (6/5) 23–66; 38.5 (17.7)				
Shahidi et al.^9^	≥ 6 m	n=45 (23/22), CNP 19–80; 43.5 (18.7)				VAS	NDI	There were no significant relationships between pain intensity and cardiovascular responses to the stressor.
Dibai-Filho et al.^15^ Brazil	≥ 3 m	n=28 (28/0), 18–45				NRSr, NRSm	NDI	No significant correlation between pain and electrical impedance. No significant correlation between disability and electrical impedance.
		28 CNP (28/0), 24 (4.19)						
Girasol et al.^14^ Brazil	≥ 3 m	n=40 (38/2), 18–45				NRS	NDI	No association between pain intensity and skin temperature. No significant correlation between disability and skin temperature.
		40 CNP (38/2), 24.31 (4.16)						
Santos-de-Araújo et al., ^10^ Brazil	≥ 3 m	n=30 (18/12), 18–45				NRSr and NRSm	NDI	No statistically significant association of NRSr with HRV indices. Statistically significant moderate negative correlation of NRSm with mean RR, mean HR, SDNN, RR Tri. Significant negative correlation between NDI and mean RRi, SDNN, RMSSD, RR Tri, and SD1.
		15 CNP (9/6) 22.46 (3.09)		15 CON (9/6) 23.73 (5.63)				
Pontes-Silva et al.^7^ Brazil	≥ 3 m	n=60 (50/10), 18–59				NRSr and NRSm	NDI	No statistically significant association between NRSr or NRSm with HRV indices (supine) in CNP group (p>0.05), except NRSm and RR Tri. Significant low-moderate negative correlation between NDI and SDNN, RMMSD, RR Tri, TINN, power HF, and SD1, SD2.
		30 CNP (26/4) 31.5 (8.4)		30 LBP (24/6) 30.6 (7.7)				

CNP: chronic neck pain; CON: control group; ANS: autonomic nervous system; SD: standard deviation; NRSr: Numerical Rating Scale At Rest; NRSm: Numerical Rating Scale At Movement; VAS: Visual Analog Scale; NDI: Neck Disability Index; HRV: heart rate variability; SDNN: square root of the mean squared differences of successive normal NN interval; PNN50: NN50 as a percentage of the total number of NN intervals; LF: low frequency; HF: high frequency; LFnorm: low frequency in normalized unit; HGT: handgrip test; MBF: muscle blood flow; Tsk: skin temperature; MST: muscle skin temperature; Evap: evaporation; EI: electrical impedance; BP: blood pressure; HR: heart rate; TINN: triangular interpolation of NN interval; RR TRI: RR triangular index; SD1: standard deviation 1; SD2: standard deviation 2; LBP: low back pain; r: correlation coefficient; p: level of significance; ꞵ: regression coefficient.

### Quality assessment

The Newcastle–Ottawa Scale (NOS) was used to assess the methodological quality of the included studies^12^. The included studies were assessed in three domains (selection, comparability, and outcomes), with a maximum score of 9. A score of 7 or above was regarded as high quality, scores ranging from 3 to 6 were considered moderate quality, and a score of 2 or below was categorized as low quality^13^. Two authors independently assessed the study quality (AA and II), and any disagreements were resolved by discussion among all authors.

## RESULTS

### Study selection

**Figure 1:**
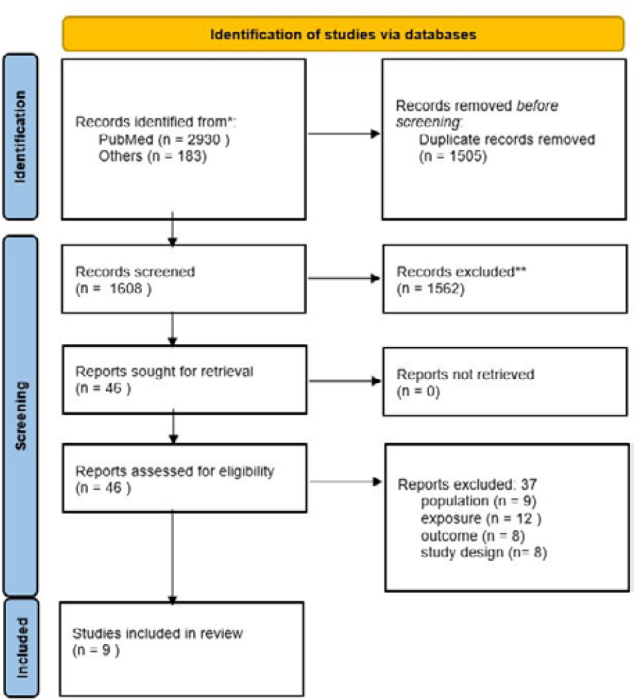
Preferred reporting items for systematic reviews and meta-analyses flowchart study selection.

A search of five databases revealed a total of 3,113 relevant articles (PubMed, 2,930; other databases, 183). After removing duplicates, 1,608 studies were processed by reviewing the titles and abstracts, and 46 studies were selected for full-text analysis. Of these, only eight met the selection criteria for inclusion in this review. The detailed PRISMA flowchart is shown in Figure 1.

### Study characteristics

Of the eight studies included, six were cross-sectional studies, while the other two studies did not explicitly mention the study design. The total population size was 440, of which 353 were female and 87 were male. All the included studies were published between 2011 and 2023.

In total, 12 autonomic tests were performed in all included studies. Heart rate variability (HRV) was used in four studies. In one study using HRV, three alternate ANS tests (electromyography, muscle blood flow, blood pressure, and heart rate fluctuation at rest and in response to handgrip test, cold pressor test, and deep breathing test) were also reported. Skin temperature alone was used in one study and with electrical impedance in another study^14,15^. Fingertip skin temperature and evaporation were measured together in one study^16^, and finally, autonomic reactivity to mental stressors was used in one study^9^.

### Results of quality assessment

The results for the methodological quality of the eligible studies are shown in Table 2. The overall score of each study ranged from five to nine out of the total nine scores. After reviewing all the studies included, four of them were considered high quality^7,10,14,17^, and four of them were of moderate quality^6,9,15,16^.

**Table 2:** Evaluation of quality assessment of included studies.

Articles	Selection (Maximum 5 stars)	Comparability (Maximum 1 star)	Outcome (Maximum 3 stars)	NOS score (Maximum 9 stars)	Quality
Hallman et al. ^6^	***	*	**	6/9	Moderate
Kang et al. ^17^	****	*	**	7/9	High
Zaprounida et al. ^16^	**	*	**	5/9	Moderate
Shahidi et al. ^9^	***	*	**	6/9	Moderate
Dibai-filho et al. ^15^	***	–	***	6/9	Moderate
Girasol et al. ^14^	****	*	***	8/9	High
Santos-de-Araújo et al. ^10^	*****	*	***	9/9	High
Pontes-silva et al. ^7^	*****	–	**	7/9	High

NOS: Newcastle-Ottawa Scale.

### Main findings

#### Association between autonomic nervous system and pain intensity

When reviewing studies assessing ANS regulation using HRV, one moderate-quality study revealed a negative correlation between resting pain intensity and basal autonomic dysregulation (particularly parasympathetic activity)^6^, whereas such a correlation at rest was not reported by two high-quality studies^7,10^. Additionally, the correlation between pain intensity and autonomic regulation has not been studied in a high-quality study^17^.

One high-quality study assessing ANS regulation using skin temperature showed no correlation between resting pain intensity and autonomic dysregulation^14^, while one moderate-quality study revealed a moderate positive correlation when symptoms were unilateral^16^. However, another moderate-quality study provided no information on its correlation with skin temperature^15^.

Furthermore, a moderate-quality study that utilized cardiovascular reflexes in response to mental stressors to assess ANS regulation reported no significant association between resting pain intensity and autonomic dysregulation^9^.

#### Association between autonomic nervous system and disability

While taking disability into account, three high-quality studies and one moderate-quality study performed HRV analysis for assessing ANS regulation, revealing a notably negative correlation of disability with reduced basal parasympathetic activity and overall autonomic dysregulation^6,7,10,17^. Conversely, two moderate-quality and one high-quality study that performed alternate ANS assessment techniques found no association with disability^14,15,16^. However, one moderate-quality study did not report a correlation between ANS regulation and disability^9^.

### DISCUSSION

This review aimed to systematically analyze the results of all available original articles that evaluated the association between ANS regulation and pain and disability in patients with CNP. After analysis, it was found that only two of the eight studies showed a significant correlation between pain intensity at rest and autonomic dysregulation^6,16^, while five of them showed no correlation^7,10,14,15^ and one did not report about correlation findings^17^. Despite the lack of correlation with pain intensity at rest, two high-quality studies have reported a correlation between pain intensity during movement and ANS dysregulation. These variations in results might be due to the heterogeneity in the duration of symptoms and ANS assessment techniques.

Zaproudina et al. reported a moderately positive correlation between fingertip skin temperature and resting pain intensity in individuals with unilateral symptoms, suggesting more of a vascular disorder than an autonomic disorder^16^. The study further investigated skin evaporation but found no significant relationship between ANS regulation and pain intensity. Interestingly, patients experiencing unilateral pain had notably higher evaporation values than controls. These variations in the skin temperature and evaporation values may be due to psychophysiological characteristics or emotional reactions, which could restrict the applicability of these instruments as diagnostic tools^16^.

Santos-de-Araújo et al. and Pontes-Silva et al. reported a noteworthy result: while pain intensity during movement showed a moderate negative correlation with parasympathetic activity, no correlation was seen during rest. This remark was made using HRV as an assessment technique^7,10^. Certain authors in their study proposed a pathophysiological explanation for this observation^6,14^: Patients with CNP tend to have higher muscular activity at rest following any neck movement. Consequently, increased muscular activity has been proposed to serve as a mediator between central or psychological consequences and the development or maintenance of muscle pain. Hence, it may be plausible that elevated muscular activity during movement is associated with autonomic dysregulation^6^.

This study revealed a stronger correlation between CNP and the parasympathetic system, possibly due to its close anatomical proximity to the cervical region and the sympathetic system’s association with the lumbar area^7,10^. In addition, pain duration plays an important role. Chronic pain initially inhibits parasympathetic activity, resulting in autonomic dysregulation. Later, it facilitated an inadequate sympathetic response to physical and mental stressors^6,9^. Consistent with this, few researchers have reported a blunted BP response to exercise in patients with CNP^18^. Similar trends have been observed in patients with whiplash-associated disorder^19^ and low back pain^20^.

When considering mental stressors, Shahidi et al. found no significant correlation between pain intensity and autonomic reactivity but observed an inverse relationship between pain chronicity and blood pressure in response to psychological stress, but not heart rate, in response to the psychological stressor^9^. This discrepancy might be due to the belief that adaptation within the ANS occurs gradually over time because of decreased baroreceptor sensitivity to chronic pain^21^. The lack of association with pain intensity might be explained by the variation in the timing of cardiovascular response measurements. However, it appears more likely that adaptations in physiological stress responses are more strongly related to the duration of symptoms than the degree of neck pain, which varies greatly from day to day^22^.

Hallman et al. demonstrated a negative correlation between disability and reduced basal parasympathetic activity and sympathetic response to physical stressors, consistent with the findings of Kang et al.^17^. Similar trends have been observed in chronic low back pain^23^. This might be due to the higher levels of mood disorders, pain severity, and sleep problems, as displayed by patients with greater levels of disability.

An important point to consider is that the majority of the population is female, and it is not surprising to comment that females are more prone to developing CNP^1^. In addition, the response to autonomic regulation can be influenced by age, measurement, time of day, humidity, temperature, sleep patterns, physical activity, and the use of medication, alcohol, and caffeine. Unfortunately, it is difficult to determine whether the results were caused by pain and disability or whether ANS dysregulation was influenced by one or more of these factors because the majority of the included studies did not indicate whether these factors were controlled.

Our study is the first systematic review to analyze the association between autonomic dysregulation and pain and disability in patients with CNP. However, some limitations were encountered during this review. First, the design of the included studies was cross-sectional; thus, the causal relationship could not be explained. Additionally, we detected large methodological heterogeneity between the studies regarding the severity of symptoms, duration of pain symptoms, assessment techniques, and control of confounding factors, compromising the findings.

## CONCLUSION

The results suggest that the higher the pain intensity and perceived disability in patients with CNP, the greater the autonomic dysregulation. Additionally, it appears that, as the duration of pain symptoms increases, the ANS becomes more provoked and becomes increasingly dysregulated. Considering the impact of this condition, further research employing larger cohorts, standardized methodologies, and more comprehensive assessments of both autonomic regulation and related clinical outcomes is required to increase the quality of evidence and guide clinical practice.
